# Supplementary material for: Quantitative CRACI reveals transcriptome-wide distribution of RNA dihydrouridine at base resolution
Source: Nat Commun. 2025 Oct 6;16:8863. doi: 10.1038/s41467-025-63918-w (PMC12501074; doi:10.1038/s41467-025-63918-w)
Supplement: Supplementary file 1 — Supplementary Information [file 41467_2025_63918_MOESM1_ESM.pdf]

Supplementary Information

Quantitative CRACI reveals transcriptome-wide distribution of RNA dihydrouridine at base resolution

Cheng-Wei Ju [#,1,2,3], Han Li [#,1,2], Bochen Jiang[1,2,4], Xuanhao Zhu[1,2], Liang Cui[5], Zhanghui Han[6], Junxi Zou[1,2], Yunzheng Liu[7], Shenghai Shen[8,9], Hardik Shah[10], Chang Ye[1, 2], Yuhao Zhong[1, 2], Ruiqi Ge[1, 2], Peng Xia[1, 2], Yiyi Ji[1, 2], Shun Liu[1,2], Fan Yang[1,2], Bei Liu[1,2], Yuzhi Xu[11], Jiangbo Wei[6,12], Li-Sheng Zhang[*,8,9], Chuan He[*,1,2,3]

1 Department of Chemistry, The University of Chicago, Chicago, IL, USA
2 Howard Hughes Medical Institute, The University of Chicago, Chicago, IL, USA
3 Pritzker School of Molecular Engineering, The University of Chicago, Chicago, IL, USA
4 School of Life Sciences & Biotechnology, Shanghai Jiao Tong University, Shanghai, China
5 Antimicrobial Resistance Interdisciplinary Research Group, Singapore-MIT Alliance for Research and Technology, Singapore, Singapore
6 Department of Chemistry, National University of Singapore, Singapore, Singapore
7 Division of Biology and Biological Engineering, California Institute of Technology, Pasadena, CA, USA
8 Division of Life Science, The Hong Kong University of Science and Technology, Kowloon, Hong Kong SAR, China
9 Department of Chemistry, The Hong Kong University of Science and Technology, Kowloon, Hong Kong SAR, China
10 Metabolomics Platform, Comprehensive Cancer Center, The University of Chicago, Chicago, IL, USA
11 Department of Chemistry, New York University, New York, NY, USA
12 Department of Biological Sciences, National University of Singapore, Singapore, Singapore
# These authors contributed equally to this work.

Email: L.-S. Zhang, zhangls@ust.hk; C. He, chuanhe@uchicago.edu.

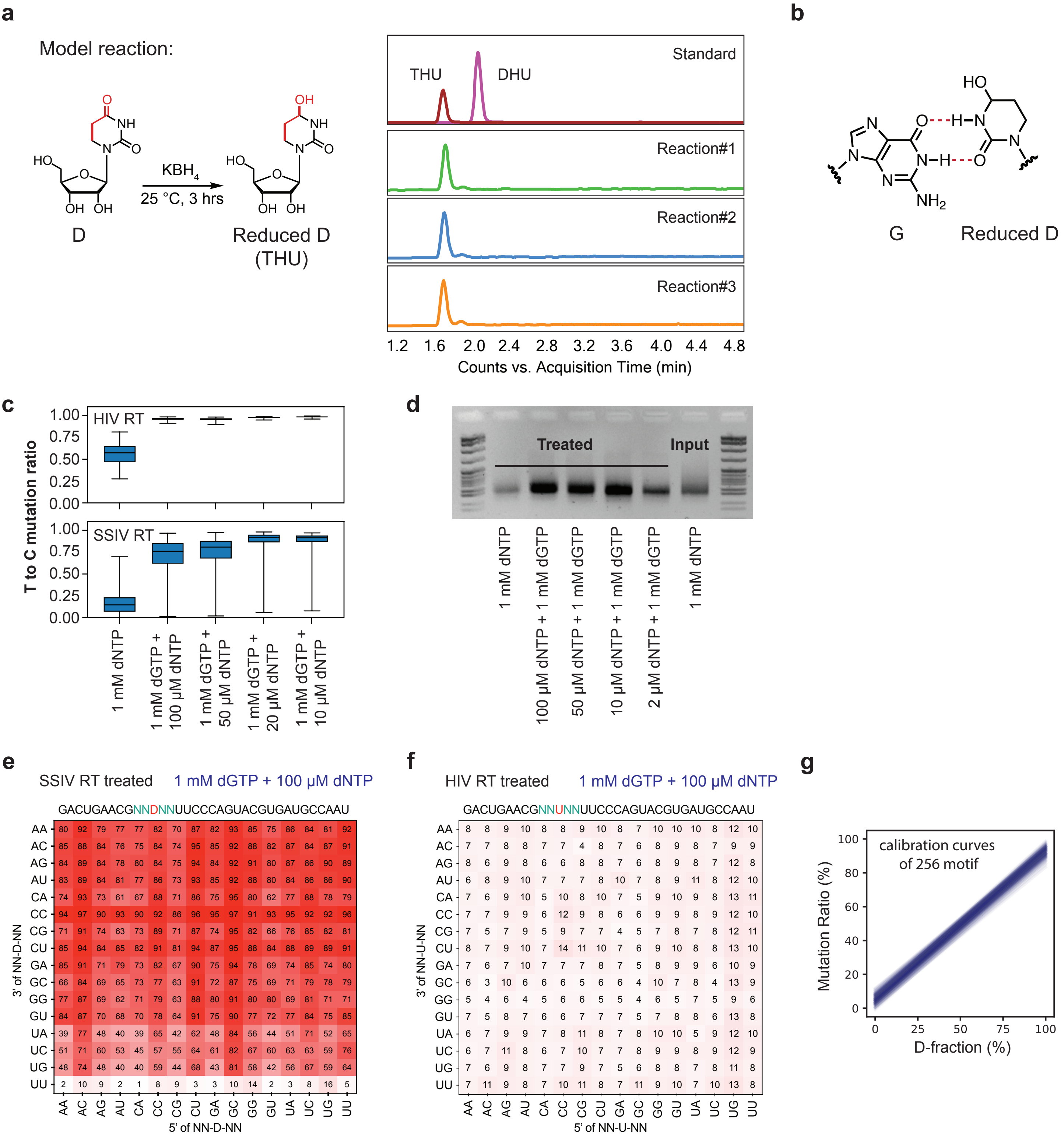
a
Model reaction:
D
KBH4
25 °C, 3 hrs
Reduced D
(THU)
Standard
THU
DHU
Reaction#1
Reaction#2
Reaction#3
1.2 1.6 2.0 2.4 2.8 3.2 3.6 4.0 4.4 4.8
Counts vs. Acquisition Time (min)
b
G
Reduced D
c
T to C mutation ratio
HIV RT
SSIV RT
1.00 0.75 0.50 0.25 0.00
1 mM dNTP
1 mM dGTP + 100 µM dNTP
1 mM dGTP + 50 µM dNTP
1 mM dGTP + 20 µM dNTP
1 mM dGTP + 10 µM dNTP
d
Treated
Input
1 mM dNTP
100 µM dNTP + 1 mM dGTP
50 µM dNTP + 1 mM dGTP
10 µM dNTP + 1 mM dGTP
2 µM dNTP + 1 mM dGTP
1 mM dNTP
e
SSIV RT treated
1 mM dGTP + 100 µM dNTP
GACUGAACGNNDNNUUCCCAGUACGUGAUGCCAAU
3' of NN-D-NN
5' of NN-D-NN
f
HIV RT treated
1 mM dGTP + 100 µM dNTP
GACUGAACGNNUNNUUCCCAGUACGUGAUGCCAAU
3' of NN-U-NN
5' of NN-U-NN
g
calibration curves of 256 motif
Mutation Ratio (%)
D-fraction (%)

Supplementary Figure 1. Optimization of RT conditions in CRACI.

a. Model reaction of the reduction from the D nucleoside to THU using KBH_4 at 25 °C for 3 hours. Liquid chromatography-tandem mass spectrometry (LC-MS/MS) chromatograms comparing the retention times of standard THU and D with products from the reduction reactions. **b.** Proposed base-pairing model between guanosine (G) and reduced D. **c.** Box plots show the misincorporation ratio observed at reduced D site of a synthetic 35-mer RNA oligo containing NNDNN under different RT conditions in CRACI (n=256 motifs). Box plots show the median (line), 25th–75th percentiles (bounds) and minimum/maximum values (whiskers). **d.** DNA gel image of CRACI libraries built with different combinations of dGTP and dNTP in the presence of HIV RT, starting with HepG2 cellular small RNA. **e.** Motif-dependent misincorporation patterns observed in CRACI using a synthetic 35-mer RNA oligo centered with NNDNN, in the presence of SSIV RT and 1 mM/100 μM dGTP/dNTP. **f.** Motif-dependent misincorporation patterns observed in CRACI using a synthetic 35-mer RNA oligo centered with NNUNN, in the presence of HIV RT and 1 mM/100 μM dGTP/dNTP. **g.** Sequence context-dependent calibration curves.

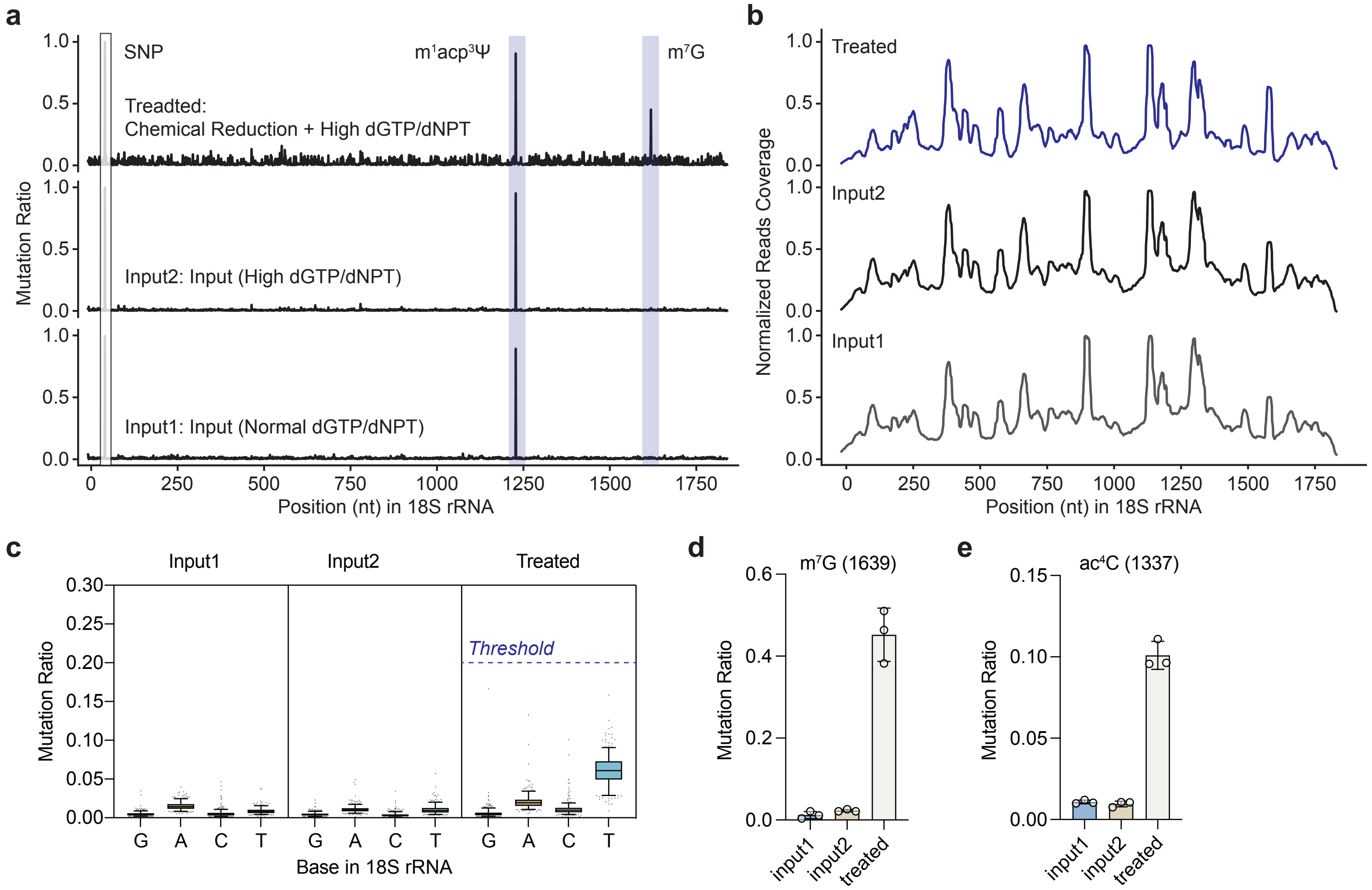

a
SNP
m¹acp³Ψ
m⁷G
Treadted:
Chemical Reduction + High dGTP/dNPT
Input2: Input (High dGTP/dNPT)
Input1: Input (Normal dGTP/dNPT)
Mutation Ratio
Position (nt) in 18S rRNA
b
Treated
Input2
Input1
Normalized Reads Coverage
Position (nt) in 18S rRNA
c
Input1
Input2
Treated
Threshold
Mutation Ratio
G A C T
Base in 18S rRNA
d
m⁷G (1639)
Mutation Ratio
input1
input2
treated
e
ac⁴C (1337)
Mutation Ratio
input1
input2
treated

Supplementary Figure 2. Evaluation of the background of CRACI using cellular 18S rRNA.

a. Mutation ratio across the 18S rRNA in input and treated samples. Known modified positions such as $m^1acp^3\Psi$ and m^7G are highlighted. The mutation ratio was calculated as the average of three biological replicates. **b.** Normalized read coverage across 18S rRNA for input and treated conditions, suggesting consistent coverage and the absence of major RT truncations. The reads coverage was calculated as the average of three biological replicates. **c.** Boxplot showing base mutation frequencies (G, A, C, T) in 18S rRNA for each sample. **d.** Mutation ratio at the m^7G1639 across different conditions. **e.** Mutation ratio at the ac^4C1337 across different conditions.

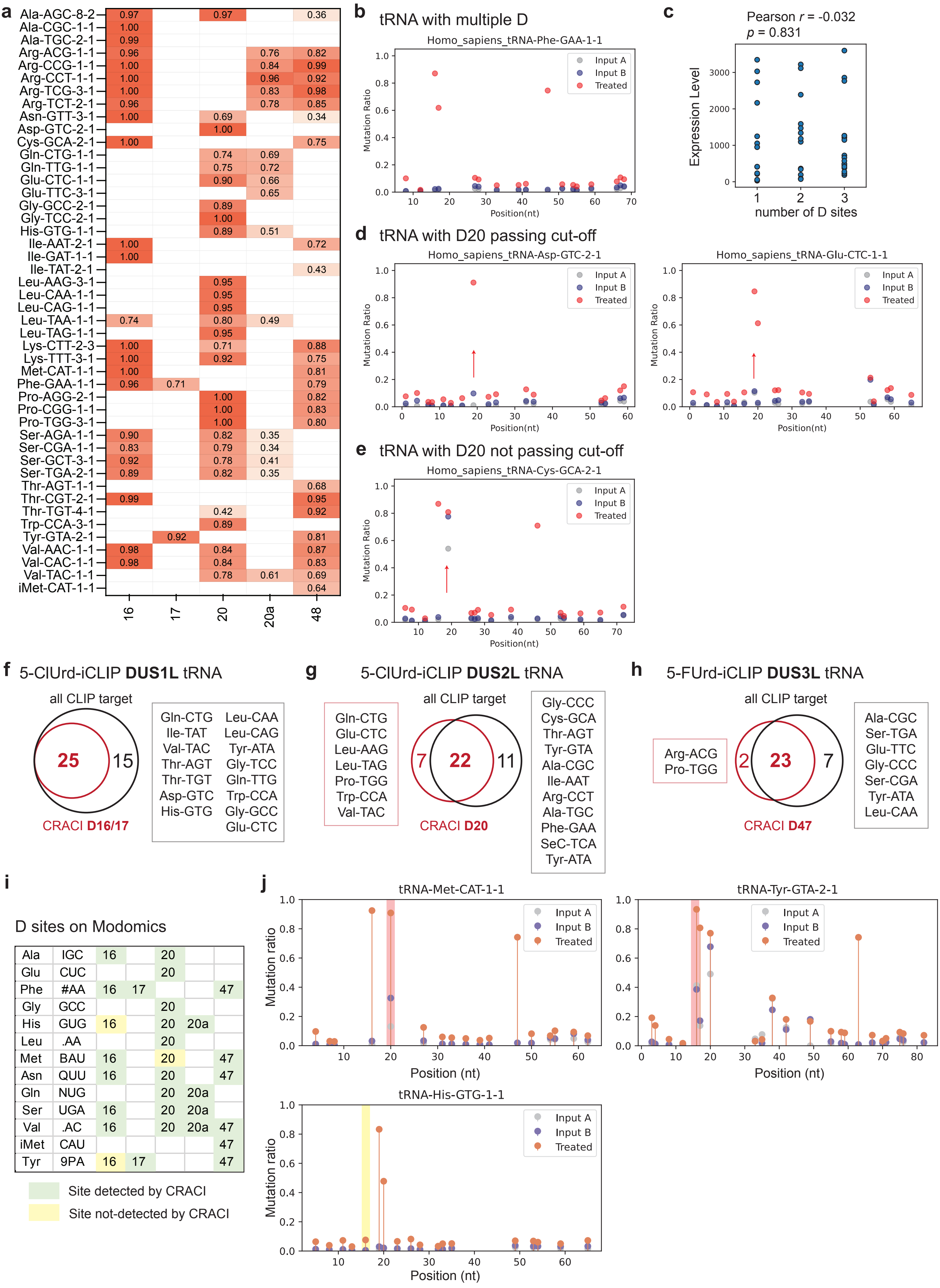
a
Ala-AGC-8-2 0.97 0.97 0.36
Ala-CGC-1-1 1.00
Ala-TGC-2-1 0.99
Arg-ACG-1-1 0.96 0.76 0.82
Arg-CCG-1-1 1.00 0.84 0.99
Arg-CCT-1-1 1.00 0.96 0.92
Arg-TCG-3-1 1.00 0.83 0.98
Arg-TCT-2-1 0.96 0.78 0.85
Asn-GTT-3-1 1.00 0.69 0.34
Asp-GTC-2-1 1.00
Cys-GCA-2-1 1.00 0.75
Gln-CTG-1-1 0.74 0.69
Gln-TTG-1-1 0.75 0.72
Glu-CTC-1-1 0.90 0.66
Glu-TTC-3-1 0.65
Gly-GCC-2-1 0.89
Gly-TCC-2-1 1.00
His-GTG-1-1 0.89 0.51
Ile-AAT-2-1 1.00 0.72
Ile-GAT-1-1 1.00
Ile-TAT-2-1 0.43
Leu-AAG-3-1 0.95
Leu-CAA-1-1 0.95
Leu-CAG-1-1 0.95
Leu-TAA-1-1 0.74 0.80 0.49
Leu-TAG-1-1 0.95
Lys-CTT-2-3 1.00 0.71 0.88
Lys-TTT-3-1 1.00 0.92 0.75
Met-CAT-1-1 1.00 0.81
Phe-GAA-1-1 0.96 0.71 0.79
Pro-AGG-2-1 1.00 0.82
Pro-CGG-1-1 1.00 0.83
Pro-TGG-3-1 1.00 0.80
Ser-AGA-1-1 0.90 0.82 0.35
Ser-CGA-1-1 0.83 0.79 0.34
Ser-GCT-3-1 0.92 0.78 0.41
Ser-TGA-2-1 0.89 0.82 0.35
Thr-AGT-1-1 0.68
Thr-CGT-2-1 0.99 0.95
Thr-TGT-4-1 0.42 0.92
Trp-CCA-3-1 0.89
Tyr-GTA-2-1 0.92 0.81
Val-AAC-1-1 0.98 0.84 0.87
Val-CAC-1-1 0.98 0.84 0.83
Val-TAC-1-1 0.78 0.61 0.69
iMet-CAT-1-1 0.64
16 17 20 20a 48
b
tRNA with multiple D
Homo_sapiens_tRNA-Phe-GAA-1-1
Input A
Input B
Treated
Mutation Ratio
Position(nt)
c
Pearson r = -0.032
p = 0.831
Expression Level
number of D sites
d
tRNA with D20 passing cut-off
Homo_sapiens_tRNA-Asp-GTC-2-1
Homo_sapiens_tRNA-Glu-CTC-1-1
e
tRNA with D20 not passing cut-off
Homo_sapiens_tRNA-Cys-GCA-2-1
f
5-ClUrd-iCLIP DUS1L tRNA
all CLIP target
25
15
CRACI D16/17
Gln-CTG Leu-CAA
Ile-TAT Leu-CAG
Val-TAC Tyr-ATA
Thr-AGT Gly-TCC
Thr-TGT Gln-TTG
Asp-GTC Trp-CCA
His-GTG Gly-GCC
Glu-CTC
g
5-ClUrd-iCLIP DUS2L tRNA
all CLIP target
Gln-CTG
Glu-CTC
Leu-AAG
Leu-TAG
Pro-TGG
Trp-CCA
Val-TAC
7
22
11
CRACI D20
Gly-CCC
Cys-GCA
Thr-AGT
Tyr-GTA
Ala-CGC
Ile-AAT
Arg-CCT
Ala-TGC
Phe-GAA
SeC-TCA
Tyr-ATA
h
5-FUrd-iCLIP DUS3L tRNA
all CLIP target
Arg-ACG
Pro-TGG
2
23
7
CRACI D47
Ala-CGC
Ser-TGA
Glu-TTC
Gly-CCC
Ser-CGA
Tyr-ATA
Leu-CAA
i
D sites on Modomics
Ala IGC 16 20
Glu CUC 20
Phe #AA 16 17 47
Gly GCC 20
His GUG 16 20 20a
Leu .AA 20
Met BAU 16 20 47
Asn QUU 16 20 47
Gln NUG 20 20a
Ser UGA 16 20 20a
Val .AC 16 20 20a 47
iMet CAU 47
Tyr 9PA 16 17 47
Site detected by CRACI
Site not-detected by CRACI
j
tRNA-Met-CAT-1-1
tRNA-Tyr-GTA-2-1
tRNA-His-GTG-1-1
Input A
Input B
Treated
Mutation ratio
Position (nt)

Supplementary Figure 3. D profiles in HepG2 ct-tRNA and mt-tRNA. a. The plot of D stoichiometry at various positions within diverse ct-tRNAs. **b.** The T→C misincorporation ratio at U sites across tRNA-Phe-GAA-1-1, as a typical example of a tRNA carrying multiple D sites. The mutation ratios were calculated as the average of two biological replicates. **c.** Relationship between the number of D sites and the expression level of tRNAs. **d.** The T→C misincorporation ratio at U sites across tRNA-Asp-GTC-2-1 and tRNA-Glu-CTC-1-1, as typical examples of previously reported D20 sites regulated by DUS2L. **e,** The T→C misincorporation ratio at U sites across tRNA-Cys-GCA-2-1, as a typical example of D20 sites that did not pass the cut-off, indicating the presence of other uridine modifications. **f.** Overlap between CRACI-detected D16/17 sites and 5-ClUrd-iCLIP targets of DUS1L. **g.** Overlap between CRACI-detected D20 sites and 5-ClUrd-iCLIP targets of DUS2L. **h.** Overlap between CRACI-detected D47 sites and 5-ClUrd-iCLIP targets of DUS3L. **i.** Table of D sites recorded in Modomics across different tRNAs. Green boxes indicate sites detected by CRACI, while yellow boxes indicate sites not detected by CRACI. **j**. The T→C misincorporation ratio at U sites in tRNA-Met-CAT-1-1 tRNA-Tyr-GTA-2-1, and tRNA-His-GTG-1-1 in HepG2. Highlighted regions correspond to Modomics recorded D sites. The mutation ratios are calculated as the average of two biological replicates.

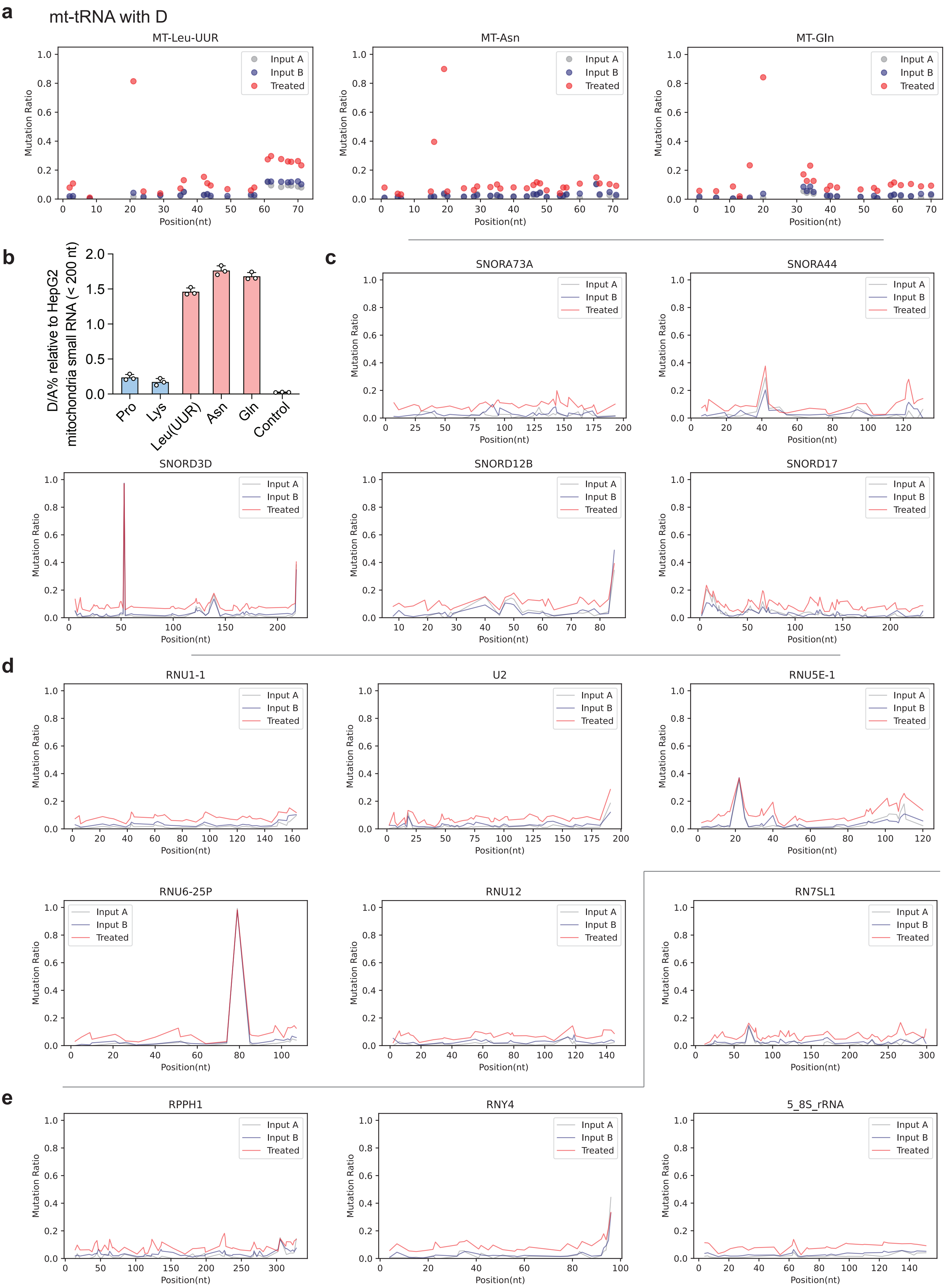
a
mt-tRNA with D
MT-Leu-UUR
MT-Asn
MT-Gln
Input A
Input B
Treated
Mutation Ratio
Position(nt)
b
D/A% relative to HepG2 mitochondria small RNA (< 200 nt)
Pro
Lys
Leu(UUR)
Asn
Gln
Control
c
SNORA73A
SNORA44
SNORD3D
SNORD12B
SNORD17
d
RNU1-1
U2
RNU5E-1
RNU6-25P
RNU12
RN7SL1
e
RPPH1
RNY4
5_8S_rRNA

Supplementary Figure 4. D is absent in human small non-coding RNAs other than tRNA. a. The T→C misincorporation ratio at U sites across several mitochondrial tRNAs in HepG2. **b.** Quantification of the D/A ratio in mitochondrial tRNAs (mt-tRNA-Pro, mt-tRNA-Lys, mt-tRNA-Leu(UUR), mt-tRNA-Asn, mt-tRNA-Gln) relative to mitochondrial small RNAs by LC-MS/MS. Probe-only samples were used as control. **c.** D is absent in snoRNAs, as shown with T→C misincorporation ratios at U sites across representative H/ACA box snoRNAs and C/D box snoRNAs, including SNORA73A, SNORA44, SNORD3D, SNORD12B, and SNORD17. **d.** D is absent in snRNAs, as shown with T→C misincorporation ratios at U sites across U1, U2, U5, U6, and U12 snRNAs. **e.** D is absent in other abundant ncRNAs, as shown with T→C misincorporation ratios at U sites across RPPH1, RNY4, 5.8S rRNA, and 7SL RNA. The mutation ratios were calculated as the average of two biological replicates.

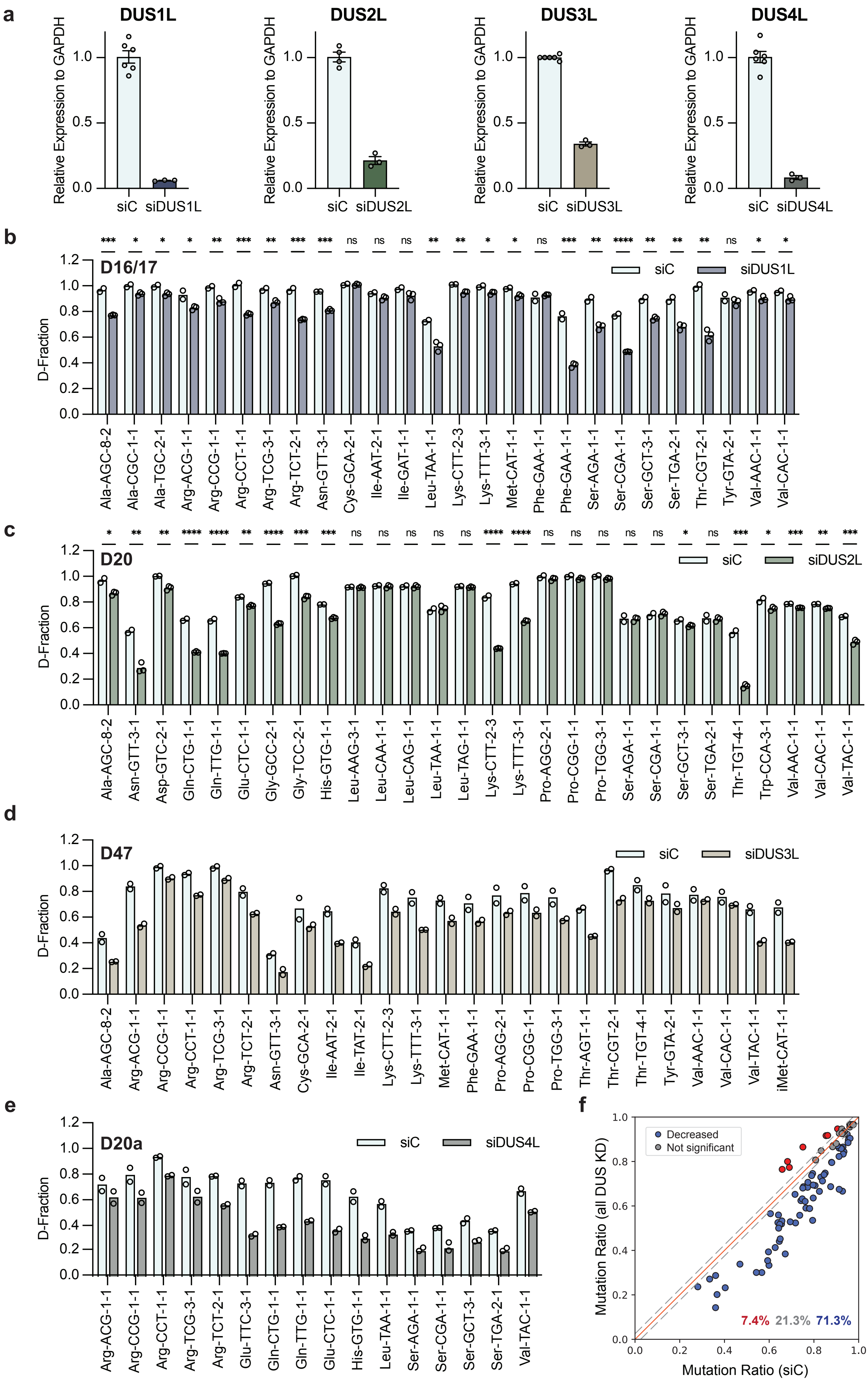

a
DUS1L
DUS2L
DUS3L
DUS4L
Relative Expression to GAPDH
0.0
0.5
1.0
siC
siDUS1L
siDUS2L
siDUS3L
siDUS4L
b
D16/17
siC
siDUS1L
D-Fraction
*** * * * ** *** ** *** *** ns ns ns ** ** * * ns *** ** **** ** ** ** ns * *
Ala-AGC-8-2
Ala-CGC-1-1
Ala-TGC-2-1
Arg-ACG-1-1
Arg-CCG-1-1
Arg-CCT-1-1
Arg-TCG-3-1
Arg-TCT-2-1
Asn-GTT-3-1
Cys-GCA-2-1
Ile-AAT-2-1
Ile-GAT-1-1
Leu-TAA-1-1
Lys-CTT-2-3
Lys-TTT-3-1
Met-CAT-1-1
Phe-GAA-1-1
Phe-GAA-1-1
Ser-AGA-1-1
Ser-CGA-1-1
Ser-GCT-3-1
Ser-TGA-2-1
Thr-CGT-2-1
Tyr-GTA-2-1
Val-AAC-1-1
Val-CAC-1-1
c
D20
siC
siDUS2L
D-Fraction
* ** ** **** **** ** **** *** *** ns ns ns ns ns **** **** ns ns ns ns ns * ns *** * *** ** ***
Ala-AGC-8-2
Asn-GTT-3-1
Asp-GTC-2-1
Gln-CTG-1-1
Gln-TTG-1-1
Glu-CTC-1-1
Gly-GCC-2-1
Gly-TCC-2-1
His-GTG-1-1
Leu-AAG-3-1
Leu-CAA-1-1
Leu-CAG-1-1
Leu-TAA-1-1
Leu-TAG-1-1
Lys-CTT-2-3
Lys-TTT-3-1
Pro-AGG-2-1
Pro-CGG-1-1
Pro-TGG-3-1
Ser-AGA-1-1
Ser-CGA-1-1
Ser-GCT-3-1
Ser-TGA-2-1
Thr-TGT-4-1
Trp-CCA-3-1
Val-AAC-1-1
Val-CAC-1-1
Val-TAC-1-1
d
D47
siC
siDUS3L
D-Fraction
Ala-AGC-8-2
Arg-ACG-1-1
Arg-CCG-1-1
Arg-CCT-1-1
Arg-TCG-3-1
Arg-TCT-2-1
Asn-GTT-3-1
Cys-GCA-2-1
Ile-AAT-2-1
Ile-TAT-2-1
Lys-CTT-2-3
Lys-TTT-3-1
Met-CAT-1-1
Phe-GAA-1-1
Pro-AGG-2-1
Pro-CGG-1-1
Pro-TGG-3-1
Thr-AGT-1-1
Thr-CGT-2-1
Thr-TGT-4-1
Tyr-GTA-2-1
Val-AAC-1-1
Val-CAC-1-1
Val-TAC-1-1
iMet-CAT-1-1
e
D20a
siC
siDUS4L
D-Fraction
Arg-ACG-1-1
Arg-CCG-1-1
Arg-CCT-1-1
Arg-TCG-3-1
Arg-TCT-2-1
Glu-TTC-3-1
Gln-CTG-1-1
Gln-TTG-1-1
Glu-CTC-1-1
His-GTG-1-1
Leu-TAA-1-1
Ser-AGA-1-1
Ser-CGA-1-1
Ser-GCT-3-1
Ser-TGA-2-1
Val-TAC-1-1
f
Decreased
Not significant
Mutation Ratio (all DUS KD)
Mutation Ratio (siC)
7.4%
21.3%
71.3%

Supplementary Figure 5. CRACI captured reduced D levels in tRNAs upon DUS depletion in HepG2 cells. a. Expression levels of four DUS enzymes, normalized by GAPDH, following 72-hour siRNA-mediated knockdown. n=6 biological replicates for siControl and n=3 biological replicates for DUS knockdown cells. **b.** D modification levels at ct-tRNA D16/D17 sites in HepG2 cells, after *DUS1L* depletion. **c.** D modification levels at ct-tRNA D20 sites in HepG2 cells, after *DUS2L* depletion. **d.** D modification levels at ct-tRNA D47 sites in HepG2 cells, after *DUS3L* depletion. **e.** D modification levels at ct-tRNA D20a sites in HepG2 cells, after *DUS4L* depletion. For b-c, n.s. indicates $p > 0.05$. *$P < 0.05$; **$P < 0.01$; ***$P < 0.001$; ****$P < 0.0001$. Two biological replicates are used for control cells, *DUS3L* knockdown cells, and *DUS4L* knockdown cells. Three biological replicates are used for *DUS1L* knockdown cells and *DUS2L* knockdown cells. **f**. Scatter plot showing the correlation of mutation ratios between control cells (siC) and combined *DUS1L/2L/3L/4L* knockdown (all DUS KD). Each dot represents a D site. Sites with significantly decreased mutation ratios upon DUS knockdown were shown in blue; non-significant sites in gray; sites with increased ratios in red. At least 70% of D sites exhibited reduced modification levels following DUS depletion. The mutation ratios for each point were calculated as the average of two biological replicates.

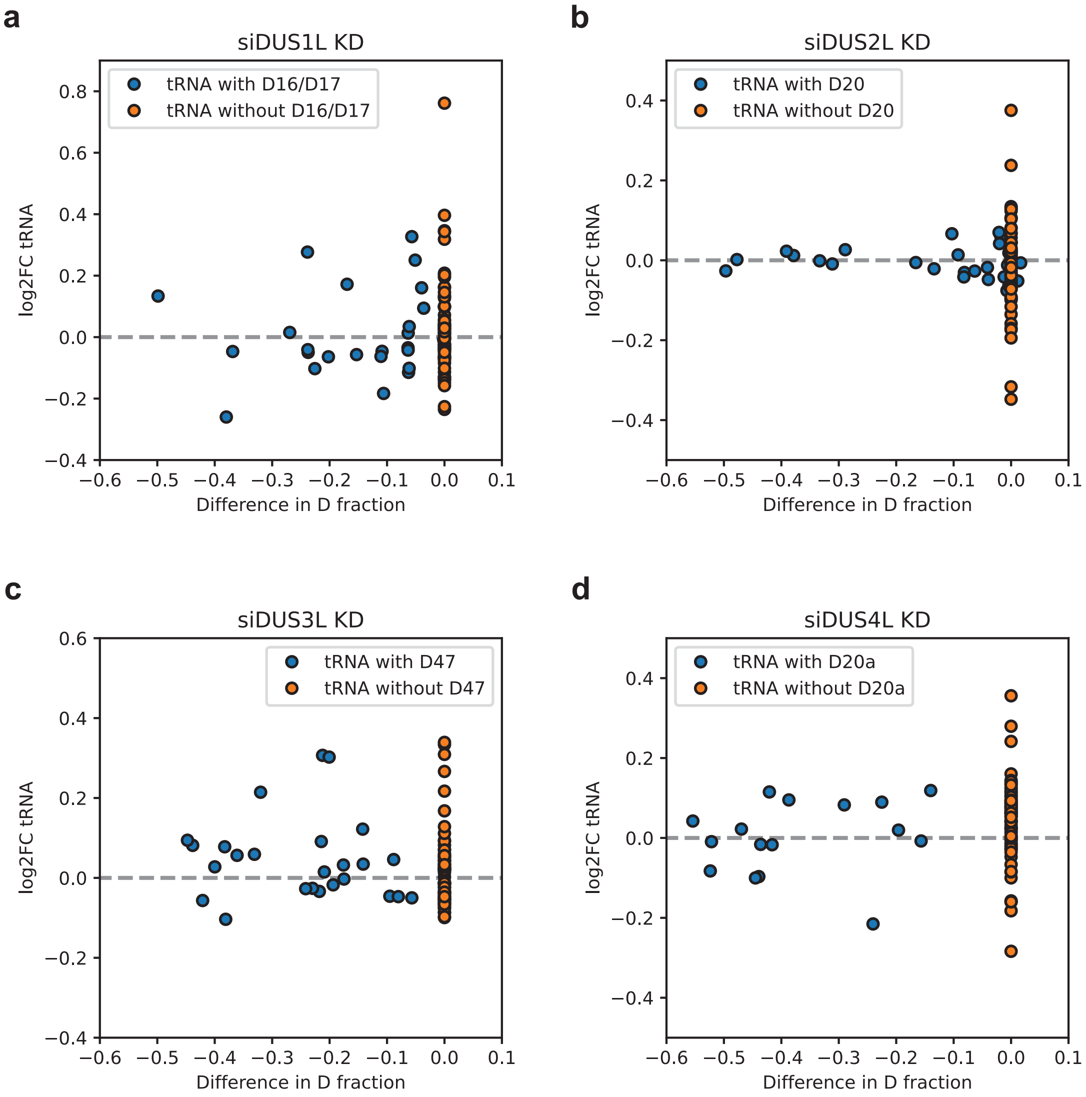
a
siDUS1L KD
tRNA with D16/D17
tRNA without D16/D17
log2FC tRNA
Difference in D fraction
b
siDUS2L KD
tRNA with D20
tRNA without D20
log2FC tRNA
Difference in D fraction
c
siDUS3L KD
tRNA with D47
tRNA without D47
log2FC tRNA
Difference in D fraction
d
siDUS4L KD
tRNA with D20a
tRNA without D20a
log2FC tRNA
Difference in D fraction

Supplementary Figure 6. The varying levels of D modifications in cytoplasmic tRNAs have a minimal impact on tRNA expression. a. Fold change in tRNA expression levels upon DUS1L depletion. tRNAs are classified as either containing D16/17 or lacking D16/17. The X-axis reflects the reduction in D stoichiometry following DUS1L depletion. **b.** Fold change in tRNA expression levels upon DUS2L depletion. tRNAs are classified as either containing D20 or lacking D20. The X-axis reflects the reduction in D stoichiometry following DUS2L depletion. **c.** Fold change in tRNA expression levels upon DUS3L depletion. tRNAs are classified as either containing D47 or lacking D47. The X-axis reflects the reduction in D stoichiometry following DUS3L depletion. **d.** Fold change in tRNA expression levels upon DUS4L depletion. tRNAs are classified as either containing D20a or lacking D20a. The X-axis reflects the reduction in D stoichiometry following DUS4L depletion. The expression levels of different RNA species were calculated as the average of at least two biological replicates.

a

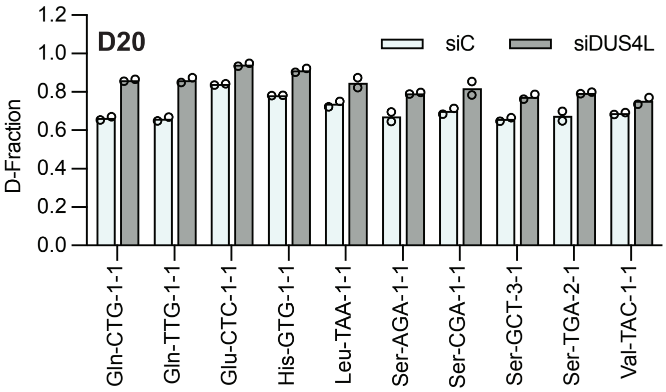

b

tRNA-Gln

GGTTCCATm^1GGTGTAATGG-**D/U-D/U**

pre-tRNA-Gln

NNN-GGTTCCATm^1GGTGTAATGG-**D/U-D/U**

tRNA-Ser

GTAGTCGTm^1GGCCGAGDGG-**D/U-D/U**

tRNA-Ser

NNN-GTAGTCGTm^1GGCCGAGDGG-**D/U-D/U**

c **d**

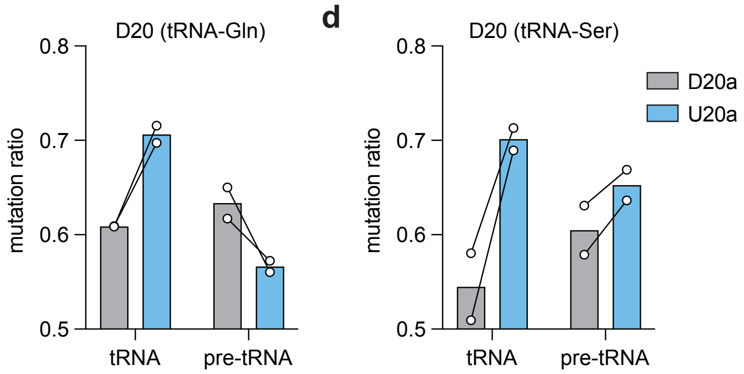

e

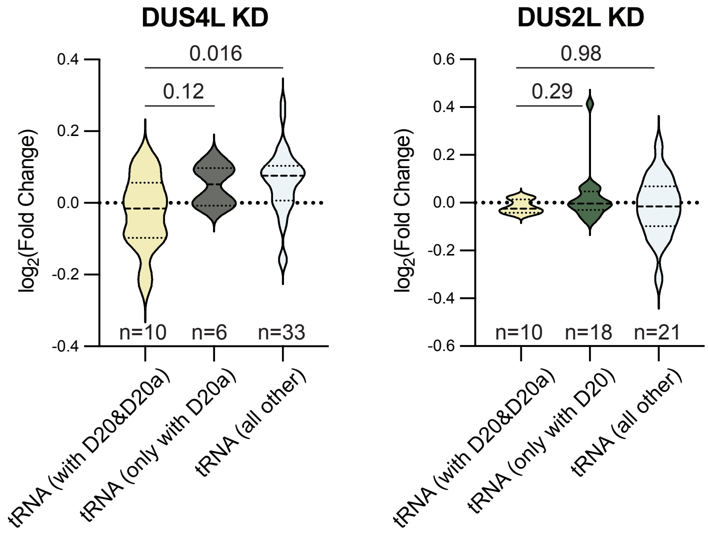

Supplementary Figure 7. D dynamics characterized by CRACI. a. D stoichiometry of selected tRNAs (‘grouped’) with D20 sites in HepG2 cells is upregulated in DUS4L-depleted cells versus the control. **b.** Schematic of the mapping methods for distinguishing pre-tRNAs and mature tRNAs. Reads containing additional nucleotides upstream of the annotated 5′ end of the tRNA were classified as originating from pre-tRNAs, while reads that start precisely at the annotated 5′ end were classified as mature tRNA reads. **c.** Comparison of mutation ratios at position 20a in tRNA-Gln (**c**) and tRNA-Ser (**d**) between mature tRNAs and pre-tRNAs. D20 levels increased during maturation in transcripts starting with U20a (blue), but remained stable in those with pre-existing D20a (gray). Each point represents one biological replicate. **e.** Comparison of tRNA expression levels in response to *DUS4L* and *DUS2L* knockdown, with tRNAs categorized as DUS targets and non-DUS targets. DUS targets are further subdivided into 'grouped' (tRNAs containing both D20 and D20a sites) and 'non-grouped’. The expression levels of different RNA species were calculated as the average of at least two biological replicates. P values from two sided unpaired t-test are shown here.

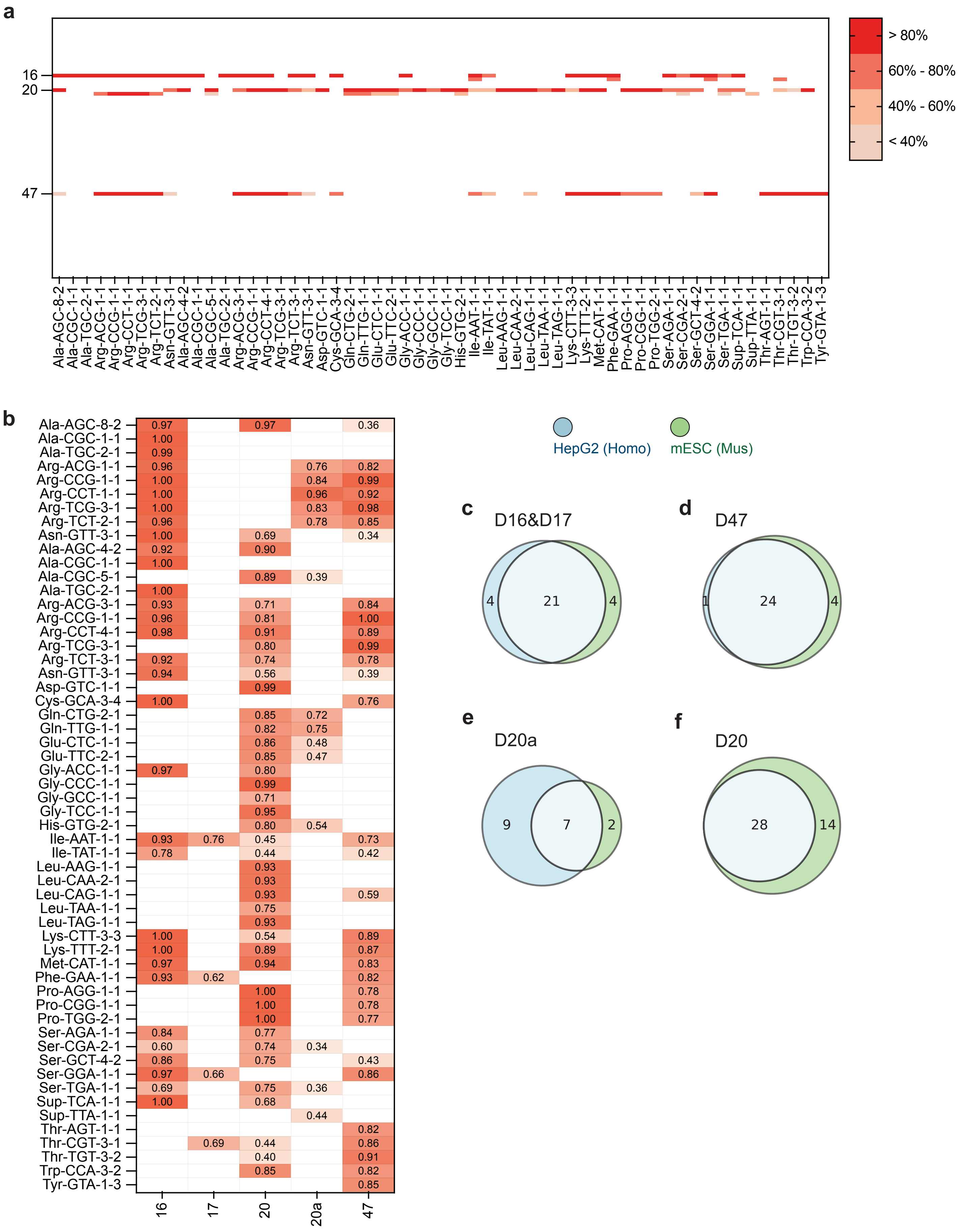

a
16
20
47
> 80%
60% - 80%
40% - 60%
< 40%
Ala-AGC-8-2
Ala-CGC-1-1
Ala-TGC-2-1
Arg-ACG-1-1
Arg-CCG-1-1
Arg-CCT-1-1
Arg-TCG-3-1
Arg-TCT-2-1
Asn-GTT-3-1
Ala-AGC-4-2
Ala-CGC-1-1
Ala-CGC-5-1
Ala-TGC-2-1
Arg-ACG-3-1
Arg-CCG-1-1
Arg-CCT-4-1
Arg-TCG-3-1
Arg-TCT-3-1
Asn-GTT-3-1
Asp-GTC-1-1
Cys-GCA-3-4
Gln-CTG-2-1
Gln-TTG-1-1
Glu-CTC-1-1
Glu-TTC-2-1
Gly-ACC-1-1
Gly-CCC-1-1
Gly-GCC-1-1
Gly-TCC-1-1
His-GTG-2-1
Ile-AAT-1-1
Ile-TAT-1-1
Leu-AAG-1-1
Leu-CAA-2-1
Leu-CAG-1-1
Leu-TAA-1-1
Leu-TAG-1-1
Lys-CTT-3-3
Lys-TTT-2-1
Met-CAT-1-1
Phe-GAA-1-1
Pro-AGG-1-1
Pro-CGG-1-1
Pro-TGG-2-1
Ser-AGA-1-1
Ser-CGA-2-1
Ser-GCT-4-2
Ser-GGA-1-1
Ser-TGA-1-1
Sup-TCA-1-1
Sup-TTA-1-1
Thr-AGT-1-1
Thr-CGT-3-1
Thr-TGT-3-2
Trp-CCA-3-2
Tyr-GTA-1-3
b
Ala-AGC-8-2 0.97 0.97 0.36
Ala-CGC-1-1 1.00
Ala-TGC-2-1 0.99
Arg-ACG-1-1 0.96 0.76 0.82
Arg-CCG-1-1 1.00 0.84 0.99
Arg-CCT-1-1 1.00 0.96 0.92
Arg-TCG-3-1 1.00 0.83 0.98
Arg-TCT-2-1 0.96 0.78 0.85
Asn-GTT-3-1 1.00 0.69 0.34
Ala-AGC-4-2 0.92 0.90
Ala-CGC-1-1 1.00
Ala-CGC-5-1 0.89 0.39
Ala-TGC-2-1 1.00
Arg-ACG-3-1 0.93 0.71 0.84
Arg-CCG-1-1 0.96 0.81 1.00
Arg-CCT-4-1 0.98 0.91 0.89
Arg-TCG-3-1 0.80 0.99
Arg-TCT-3-1 0.92 0.74 0.78
Asn-GTT-3-1 0.94 0.56 0.39
Asp-GTC-1-1 0.99
Cys-GCA-3-4 1.00 0.76
Gln-CTG-2-1 0.85 0.72
Gln-TTG-1-1 0.82 0.75
Glu-CTC-1-1 0.86 0.48
Glu-TTC-2-1 0.85 0.47
Gly-ACC-1-1 0.97 0.80
Gly-CCC-1-1 0.99
Gly-GCC-1-1 0.71
Gly-TCC-1-1 0.95
His-GTG-2-1 0.80 0.54
Ile-AAT-1-1 0.93 0.76 0.45 0.73
Ile-TAT-1-1 0.78 0.44 0.42
Leu-AAG-1-1 0.93
Leu-CAA-2-1 0.93
Leu-CAG-1-1 0.93 0.59
Leu-TAA-1-1 0.75
Leu-TAG-1-1 0.93
Lys-CTT-3-3 1.00 0.54 0.89
Lys-TTT-2-1 1.00 0.89 0.87
Met-CAT-1-1 0.97 0.94 0.83
Phe-GAA-1-1 0.93 0.62 0.82
Pro-AGG-1-1 1.00 0.78
Pro-CGG-1-1 1.00 0.78
Pro-TGG-2-1 1.00 0.77
Ser-AGA-1-1 0.84 0.77
Ser-CGA-2-1 0.60 0.74 0.34
Ser-GCT-4-2 0.86 0.75 0.43
Ser-GGA-1-1 0.97 0.66 0.86
Ser-TGA-1-1 0.69 0.75 0.36
Sup-TCA-1-1 1.00 0.68
Sup-TTA-1-1 0.44
Thr-AGT-1-1 0.82
Thr-CGT-3-1 0.69 0.44 0.86
Thr-TGT-3-2 0.40 0.91
Trp-CCA-3-2 0.85 0.82
Tyr-GTA-1-3 0.85
16
17
20
20a
47
HepG2 (Homo)
mESC (Mus)
c
D16&D17
4
21
4
d
D47
1
24
4
e
D20a
9
7
2
f
D20
28
14

Supplementary Figure 8. D profiles in *Mus musculus* (mESC) tRNAs, revealed by CRACI. a. Heatmap showing D modification stoichiometry at high-confidence D sites in mESC ct-tRNAs. A representative tRNA isoform in each tRNA class is shown. **b.** CRACI uncovers D stoichiometry at various positions within diverse mESC ct-tRNAs. (**c-f**) Venn diagrams illustrating the overlap of D sites in human (HepG2) and mouse (mESC) ct-tRNAs: (**c**) D16/17, (**d**) D47, (**e**) D20a, and (**f**) D20. The D fraction of each site was calculated as the average of two biological replicates.

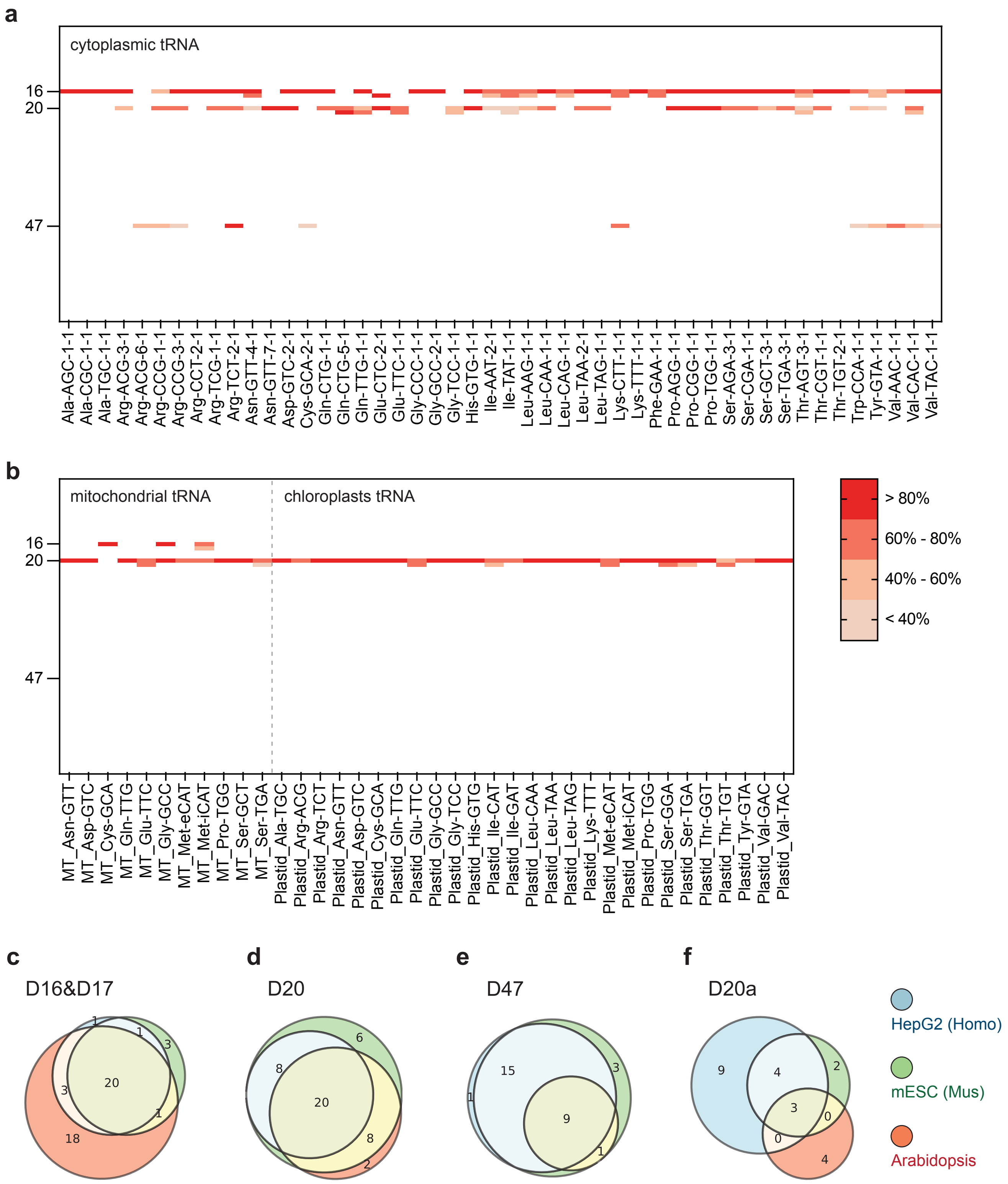

a
cytoplasmic tRNA
16
20
47
Ala-AGC-1-1
Ala-CGC-1-1
Ala-TGC-1-1
Arg-ACG-3-1
Arg-ACG-6-1
Arg-CCG-1-1
Arg-CCG-3-1
Arg-CCT-2-1
Arg-TCG-1-1
Arg-TCT-2-1
Asn-GTT-4-1
Asn-GTT-7-1
Asp-GTC-2-1
Cys-GCA-2-1
Gln-CTG-1-1
Gln-CTG-5-1
Gln-TTG-1-1
Glu-CTC-2-1
Glu-TTC-1-1
Gly-CCC-1-1
Gly-GCC-2-1
Gly-TCC-1-1
His-GTG-1-1
Ile-AAT-2-1
Ile-TAT-1-1
Leu-AAG-1-1
Leu-CAA-1-1
Leu-CAG-1-1
Leu-TAA-2-1
Leu-TAG-1-1
Lys-CTT-1-1
Lys-TTT-1-1
Phe-GAA-1-1
Pro-AGG-1-1
Pro-CGG-1-1
Pro-TGG-1-1
Ser-AGA-3-1
Ser-CGA-1-1
Ser-GCT-3-1
Ser-TGA-3-1
Thr-AGT-3-1
Thr-CGT-1-1
Thr-TGT-2-1
Trp-CCA-1-1
Tyr-GTA-1-1
Val-AAC-1-1
Val-CAC-1-1
Val-TAC-1-1
b
mitochondrial tRNA
chloroplasts tRNA
16
20
47
MT_Asn-GTT
MT_Asp-GTC
MT_Cys-GCA
MT_Gln-TTG
MT_Glu-TTC
MT_Gly-GCC
MT_Met-eCAT
MT_Met-iCAT
MT_Pro-TGG
MT_Ser-GCT
MT_Ser-TGA
Plastid_Ala-TGC
Plastid_Arg-ACG
Plastid_Arg-TCT
Plastid_Asn-GTT
Plastid_Asp-GTC
Plastid_Cys-GCA
Plastid_Gln-TTG
Plastid_Glu-TTC
Plastid_Gly-GCC
Plastid_Gly-TCC
Plastid_His-GTG
Plastid_Ile-CAT
Plastid_Ile-GAT
Plastid_Leu-CAA
Plastid_Leu-TAA
Plastid_Leu-TAG
Plastid_Lys-TTT
Plastid_Met-eCAT
Plastid_Met-iCAT
Plastid_Pro-TGG
Plastid_Ser-GGA
Plastid_Ser-TGA
Plastid_Thr-GGT
Plastid_Thr-TGT
Plastid_Tyr-GTA
Plastid_Val-GAC
Plastid_Val-TAC
> 80%
60% - 80%
40% - 60%
< 40%
c
D16&D17
1
1
3
3
20
1
18
d
D20
6
8
20
8
2
e
D47
15
3
1
9
1
f
D20a
9
4
2
3
0
0
4
HepG2 (Homo)
mESC (Mus)
Arabidopsis

Supplementary Figure 9. D profiles in *Arabidopsis Thaliana* tRNAs, revealed by CRACI. a. Heatmap showing modification stoichiometry at high-confidence D sites in *Arabidopsis Thaliana* ct-tRNAs. A representative tRNA isoform in each tRNA class is shown. **b.** Heatmap showing modification stoichiometry at high-confidence D sites in *Arabidopsis Thaliana* mt-tRNAs and chloroplasts tRNAs. A representative tRNA isoform in each tRNA class is shown. (**c-f**) Venn diagrams illustrating the overlap of D sites in ct-tRNAs from human (HepG2), mouse (mESC) and plant (*Arabidopsis Thaliana*): (**c**) D16/17, **d**) D20, (**e**) D47, and (**f**) D20a. The D fraction of each site was calculated as the average of two biological replicates.

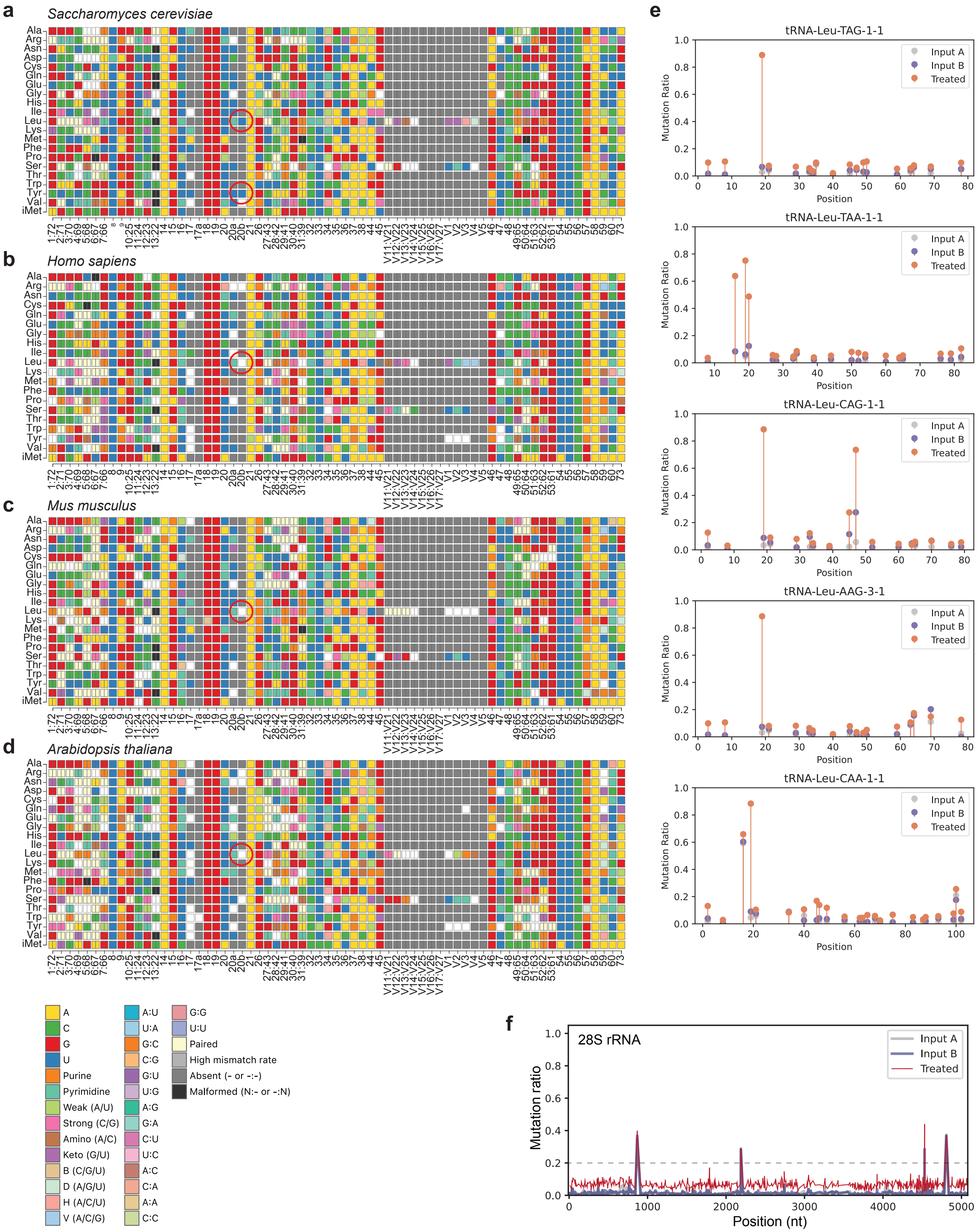
a
Saccharomyces cerevisiae
b
Homo sapiens
c
Mus musculus
d
Arabidopsis thaliana
Ala
Arg
Asn
Asp
Cys
Gln
Glu
Gly
His
Ile
Leu
Lys
Met
Phe
Pro
Ser
Thr
Trp
Tyr
Val
iMet
1:72 2:71 3:70 4:69 5:68 6:67 7:66 8 9 10:25 11:24 12:23 13:22 14 15 16 17 17a 18 19 20 20a 20b 21 26 27:43 28:42 29:41 30:40 31:39 32 33 34 35 36 37 38 44 45 V11:V21 V12:V22 V13:V23 V14:V24 V15:V25 V16:V26 V17:V27 V1 V2 V3 V4 V5 46 47 48 49:65 50:64 51:63 52:62 53:61 54 55 56 57 58 59 60 73
A
C
G
U
Purine
Pyrimidine
Weak (A/U)
Strong (C/G)
Amino (A/C)
Keto (G/U)
B (C/G/U)
D (A/G/U)
H (A/C/U)
V (A/C/G)
A:U
U:A
G:C
C:G
G:U
U:G
A:G
G:A
C:U
U:C
A:C
C:A
A:A
C:C
G:G
U:U
Paired
High mismatch rate
Absent (- or -:-)
Malformed (N:- or -:N)
e
tRNA-Leu-TAG-1-1
tRNA-Leu-TAA-1-1
tRNA-Leu-CAG-1-1
tRNA-Leu-AAG-3-1
tRNA-Leu-CAA-1-1
Mutation Ratio
Position
Input A
Input B
Treated
f
28S rRNA
Mutation ratio
Position (nt)
Input A
Input B
Treated

Supplementary Figure 10. D profiles at tRNA position 20b and in HepG2 rRNA.
a–d, Isotype-specific consensus sequence features of tRNAs in *Saccharomyces cerevisiae* (**a**), *Homo sapiens* (**b**), *Mus musculus* (**c**), and *Arabidopsis thaliana* (**d**), obtained from the tRNAviz database[56]. Potentially abundant U20b sites are marked with red circles. **e**. The T→C misincorporation ratio at U sites in multiple tRNA-Leu isodecoders (TAG, TAA, CAG, AAG, and CAA) from HepG2 cells. The D fraction of each site was calculated as the average of two biological replicates. **f.** Misincorporation signatures revealed by CRACI across 28S rRNA, confirming the absence of false-positive D sites in long RNAs. The D fraction of each site was calculated as the average of three biological replicates.

Supplementary Note
Library Preparation for CRACI

A. RNA Fragmentation.

Timing: ~30 mins

1. Dilute **~100** ng RNA samples in 10 µl of water, preheat a PCR block to 70 °C and move RNA samples to a PCR tube. Add a premix stock of 1 ul 10X RNA Fragmentation Reagents (catalog no. AM8740, Invitrogen) and 4 ul RNase-free water in to the RNA samples. Then mix well by pipetting.

Prepare the fragmentation reaction as follow:

Reagents	Volume (µL)
RNA	10
10X RNA Fragmentation Reagents	1
RNase-free water	4

Note: If there are multiple samples, it is recommended to premix the 10X RNA Fragmentation Reagents and RNase-free water and add 5 ul premixed stock each sample.

2. Heat the RNA samples to 70 °C for 14 min.

3. Purify fragmented RNA with a Oligomer Clean and Concentrator (catalog no. D4060, Zymo Research) according to the manufacturer's protocol and elute with 11 ul RNase-free water to get RNA in 10 ul RNase-free water.

Pause point: The eluted RNA (10 µl) can be stored at –80 °C for ~7 days.

B. End repair

Timing: ~3 hours

4. Prepare a stock containing 12.5 ul RNase-free water + 3 ul 10X PNK buffer (catalog no. B0201S, New England BioLabs) + 1.5 uL SUPERase•In™ (catalog no. AM2694, Invitrogen) with a final volume of 17 µL. Add premixed stock into 10 µl RNA samples from Step 3. Mix the stock well. Then add 3uL T4 PNK (catalog no. EK0032, Thermo Fisher Scientific) into the stock. Mix the stock well again.

Prepare the end repair reaction as follow:

Reagents	Volume (µL)
RNA	10
10X PNK reaction buffer	3
SUPERase•In™	1.5
RNase-free water	12.5
T4 PNK	3

Note: Mix the stock well is important for multiple samples. For the first-time mix (before adding T4 PNK), 10 times pipetting is recommended. For the second-time mix (after adding T4 PNK), 20 times pipetting is recommended.

5. Heat the samples at 37 °C for 45 min.

6. After finishing the reaction, then 1.5 μl T4 PNK, mix the stock well. Then 1.5 μl 10 mM ATP were added. Mix the stock well.

Reagents	Volume (μL)
RNA	30
T4 PNK	1.5
10 mM ATP	1.5

Note: For the first-time mix, 10 times pipetting is recommended. 20 times pipetting is recommended for the second-time mix.

6. Heat the samples at 37 °C for 45 min

7. Purify samples with Oligomer Clean and Concentrator (catalog no. D4060, Zymo Research) and elute with 11 ul RNase-free water to get 3′-repaired and 5′- phosphorylated RNA in 10 ul RNase-free water.

Pause point: The eluted RNA (10 μl) can be stored at –80 °C for ~7 days.

C. 3′-Adaptor ligation

Timing: ~14 hours

8. 10 μl 3′-repaired and 5′-phosphorylated RNA fragments were incubated with 1.5 μl 10 μM RNA 3′ SR Adapter (5′App-NNNNNATCACG AGATCGGAAGAGCACACGTCT-3SpC3, with ATCACG as the inline barcode) at 70 °C for 2 mins and placed immediately on ice.

Note: Placed immediately on ice is important.

9. Then, prepare a stock containing 2.5 μl 10× T4 RNA Ligase Reaction Buffer (catalog no. M0373L, New England BioLabs), 7.5 μl PEG8000 (50%), 1 μl SUPERase•In RNase Inhibitor (catalog no. AM2694, Invitrogen). Add the stock into the RNA–adapter mixture. Mix the samples well by pipetting 20 times. Then add 2 μl T4 RNA Ligase 2 truncated KQ (catalog no. M0373L, New England BioLabs). Mix the samples well again by pipetting 20 times.

Note: Mix the stock well is important if there are multiple samples. 20 times pipetting is required for two times mixing in this step due to the high viscosity of PEG8000. In this step, it is acceptable for adding ligase enzyme T4 RNA ligase 2 truncated KQ to all samples and then starting mixing everything.

Reagents	Volume (μL)
RNA and linker mixture	11
10X T4 RNA Ligase Reaction Buffer	2.5
50% PEG8000	7.5
SUPERase•In™	1
T4 RNA ligase 2 truncated KQ	2

10. The reaction was incubated at 25 °C for 2 h followed by 16 °C for 10 h.

11. After the reation, the reaction was further diluted to 47 µl with nuclease-free water, and add 2 µl 5′-deadenylase (catalog no. M0331S, New England BioLabs). Mix the samples well by pipetting 20 times.

Note: To avoid enzyme inactivation, do not mix the 5′-deadenylase with nuclease-free water as stock.

Reagents	Volume (µL)
Reaction Mixture	24
RNase-free water	23
2 µl 5′-deadenylase	2

12. Following, incubate the samples at 30 °C for 30 min to remove the excessive adapters .

13. After finishing the reaction, then adding 1 µl RecJf (catalog no. M0264L, New England BioLabs) for ssDNA digestion. Mix the samples well by pipetting 20 times.

Reagents	Volume (µL)
Reaction Mixture	49
RecJf	1

14. Heat the samples at 37 °C for 30 min.

15. Purify samples with a RNA Clean and Concentrator (catalog no. R1017, Zymo Research) according to the manufacturer's protocol and elute with 11 ul RNase-free water to get 3′-end-ligated RNA in 10 ul RNase-free water.

Pause point: The eluted RNA (10 µl) can be stored at –80 °C for ~7 days.

D. 5′-Adaptor ligation

Timing: ~10 hours

16. The purified RNA was incubated with 1.2 µl 10 µM 5′ SR Adapter (5′-GUUCAGAGUUCUACAGUCCGACGAUC-3′) at 70 °C for 2 mins and placed immediately on ice.

Note: Placed immediately on ice is important.

17. Then prepare a stock containing 2.5 µl 10× T4 RNA ligase reaction buffer, 1.0 µl 25 mM ATP, 10 µl PEG8000 (50%). Add the stock into the RNA–adapter mixture and mix well by pipetting for 20 times. Then, 1 µl T4 RNA Ligase 1 (high concentration, catalog no. M0437M, New England BioLabs) were added to the mixture. Mix well again by pipetting 20 times.

Note: Add ligase enzyme to one sample followed by mixing this sample immediately. **Mix each sample one by one**. Do not add ligase enzyme to all samples and then start mixing everything. 20 times pipetting is required for two times mixing in this step due to the high viscosity of PEG8000.

Reagents	Volume (μL)
RNA–adapter mixture	11
10X T4 RNA Ligase Reaction Buffer	2.5
50% PEG8000	10
25 mM ATP	1
T4 RNA Ligase 1 (high concentration)	1

18. The reaction was incubated at 25 °C for 8 h,

19. Purify samples by RNA Clean and Concentrator (catalog no. R1017, Zymo Research) according to the manufacturer's protocol and elute with 13 ul RNase-free water to get RNA in 12 ul RNase-free water.

Pause point: The eluted RNA (**12 μl**) can be stored at –80 °C for ~7 days.

E. KBH_4 Treatment

Timing: ~4 hours

20. Before KBH_4 treatment, a **2 μl aliquot of the purified RNA was saved for "*Input1*" library construction,** a **2 μl aliquot of the purified RNA was saved for "*Input2*" library construction.** The "*Input1*", ""*Input2*" samples were diluted to 10 μl with RNase-free water and storge at -80 °C.

21. The rest 8 μl RNA was mixed with 40 μl freshly prepared KBH_4 (1 M KBH_4, prepared by dissolving 54 mg KBH_4 in 1000 μl RNase-free water)

Reagents	Volume (μL)
RNA	8
1M KBH_4	40

22. Incubated the reaction mixture at 25 °C for 3 h.

23. Purify samples by RNA Clean and Concentrator (catalog no. R1017, Zymo Research) according to the manufacturer's protocol and elute with 11 ul RNase-free water to get RNA in 10 ul RNase-free water.

F. Reverse transcription

Timing: ~2 hours

24. For 'Input1', 'Input2' and 'Treated' samples (10 μl per sample) were mixed with 1.0 μl 2.0 μM SR RT primer (5′- AGACGTGTGCTCTTCCGATCT -3′) at 65 °C for 2 mins and moved immediately onto ice.

Note: Placed immediately on ice is important.

25. To the RNA-primer mixture, a stock with 2μl RNase-free water, 2 μl 10x AMV Buffer (New England BioLabs, cat. no. B0277AVIAL), 2 μl 1 mM dNTP Solution Mix (dilute from 10 mM dNTP Solution Mix,

New England BioLabs), 2 µl 10mM dGTP (dilute from 100 mM dGTP Solution, New England BioLabs), 1 µl RNaseOUT Recombinant Ribonuclease Inhibitor (catalog no. 10777019, Thermo Scientific) and 2 µl HIV RT (Worthington Biochemical, catalog no. LS05003) was added.

Reagents for "Input1" samples	Volume (µL)
RNA-primer mixture	11
RNase-free water	2
10× AMV Buffer	2
1 mM dNTP Solution Mix	2
RNaseOUT Recombinant Ribonuclease Inhibitor	1
HIV RT	2

Reagents for ""Input2" and "Treated" samples	Volume (µL)
RNA-primer mixture	11
10× AMV Buffer	2
1 mM dNTP Solution Mix	2
10 mM dGTP	2
RNaseOUT Recombinant Ribonuclease Inhibitor	1
HIV RT	2

The reaction was mixed well and incubated at 37 °C for 1 h.
Note: Mix the stock well is important for multiple samples.

Pause point: The RT product can be stored at 4 °C overnight, it is not recommended to storge for a longer time.

26. After the reaction, 1 µl RNase H (catalog no. M0297L, New England BioLabs) was added. Mix well by pipetting.

Reagents	Volume (µL)
Reaction Mixture	20
RNaseH	1

27. The reaction should be incubated at 37 °C for 20 min, and then heat at 70 °C for 5 min.

28. Purify cDNA samples with DNA Clean and Concentrator (catalog no. D4003, Zymo Research). Add a 7× volume (147 µl) of DNA binding buffer, mix by pipetting. Wash the columns according to the manufacturer's protocol. 21 ul RNase-free water was used in elution and cDNA in 20ul RNase-free water can be obtained.

Pause point: The eluted cDNAs (20 µl) were stored at –80 °C for years.

G. PCR Amplification

Timing: ~4 hours

29. 5 µl cDNA obtained from the step 31 was used for each 15-cycle PCR amplification reaction, which was performed by adding 1 ul indexed primers (from New England BioLabsNext Multiplex

Oligos for Illumina) with a stock contained: 1 ul SR Primer for Illumina (New England BioLabs), 25 ul LongAmp (catalog no. M0287S, New England BioLabs) Master Mix and 18 ul RNase-free water.

Note: To avoid mistakes in multiple samples, it is recommended to add indexed primers into PCR tubes before the addition of cDNA.

30. Perform PCR on the samples from Step 32.

Reagents	Volume (μL)
cDNA	5
indexed primers	1
SR Primer for Illumina	1
LongAmp Master Mix	25
RNase-free water	18

**cycle number**	**Denature**	**Anneal**	**Extend**
1	94 °C 45s	62 °C 15s	70 °C 30s
2-14	94 °C 15s	62 °C 15s	70 °C 30s
15	94 °C 15s	62 °C 15s	70 °C 5 mins

31. After PCR amplification, add 10 ul Gel Loading Dye (6x, catalog no.B7021S, New England BioLabs) to each well and mix well by pipetting.

32. All libraries were purified on a 3.5% low melting point agarose gel and run them at 90 V for ~45 mins. pBR322 DNA-MspI Digest (catalog no. E7323A, New England BioLabs) is used as marker.

33. Recovery DNA from gel by MinElute Gel Extraction Kit (catalog no.28604, QIAGEN) according to the manufacturer's protocol and elute with 15 ul RNase-free water.
