# Supplementary material for: Quantitative CRACI reveals transcriptome-wide distribution of RNA dihydrouridine at base resolution
Source: Nat Commun. 2025 Oct 6;16:8863. doi: 10.1038/s41467-025-63918-w (PMC12501074; doi:10.1038/s41467-025-63918-w)
Supplement: Supplementary file 2 — Description of Additional Supplementary Information [file 41467_2025_63918_MOESM2_ESM.pdf]

Supplementary Data 1. Calibration curves of D ratio and mutation ratio derived from CRACI, analyzed under varying D ratios and different sequence motifs

Supplementary Data 2. Annotation, motif analysis, and quantification of D modification sites identified by CRACI in tRNA from human HepG2 cells. P-values from unpaired two-sided t-tests are shown here.

Supplementary Data 3. Annotation, motif analysis, and quantification of D modification sites identified by CRACI in tRNA from siControl and siDUS knockdown human HepG2 cells. P-values from unpaired two-sided t-tests are shown here.

Supplementary Data 4. Annotation, motif analysis, and quantification of D modification sites identified by CRACI in tRNA from mESCs. P-values from unpaired two-sided t-tests are shown here.

Supplementary Data 5. Annotation, motif analysis, and quantification of D modification sites identified by CRACI in tRNA from Arabidopsis thaliana seedlings. P-values from unpaired two-sided t-tests are shown here.

Supplementary Data 6. tRNA sequences from Arabidopsis thaliana mitochondria and plastids with observed D sites highlighted in brackets.

Supplementary Data 7. Annotation, motif analysis, and quantification of D modification sites identified by CRACI in HepG2 mRNA based on IVT RNA. P-values from unpaired two-sided t-tests (p) and binomial test (p_binom_test) are shown here .

Supplementary Data 8. Sequences of RT-qPCR primers, CRACI adapters, and spike-in oligos.
